# Therapeutic effect and transcriptome-methylome characteristics of METTL3 inhibition in liver hepatocellular carcinoma

**DOI:** 10.1186/s12935-023-03096-1

**Published:** 2023-11-27

**Authors:** Qingbin Liu, Jingjing Qi, Weiyang Li, Xinchen Tian, Jiaqi Zhang, Fen Liu, Xiulian Lu, Hengchang Zang, Chenqiao Liu, Changlin Ma, Yong Yu, Shulong Jiang

**Affiliations:** 1 Clinical Medical Laboratory Center, Jining First People’s Hospital, Shandong First Medical University, Jining, 272000 People’s Republic of China; 2 Cisen Pharmaceutical Co. Ltd, Jining, 272000 China; 3 School of Pharmaceutical Sciences, Cheeloo College of Medicine, Shandong University, Jinan, 250100 Shandong China; 4 Johannes Kepler University Linz, Altenberger Strasse 69, 4040 Linz, Austria; 5 School of Biological Science, Jining Medical University, Rizhao, Shandong China; 6 Hepatobiliary Surgery Department, Jining First People’s Hospital, Shandong First Medical University, Jining, 272000 China

**Keywords:** METTL3, m^6^A, SMAD, MAPK, LIHC

## Abstract

Methyltransferase-like 3 (METTL3) is the key subunit of methyltransferase complex responsible for catalyzing N6-methyladenosine (m^6^A) modification on mRNA, which is the most prevalent post-transcriptional modification in eukaryotes. In this study, we utilized online databases to analyze the association between METTL3 expression and various aspects of tumorigenesis, including gene methylation, immunity, and prognosis. Our investigation revealed that METTL3 serves as a prognostic marker and therapeutic target for liver hepatocellular carcinoma (LIHC). Through experimental studies, we observed frequent upregulation of METTL3 in LIHC tumor tissue and cells. Subsequent inhibition of METTL3 using a novel small molecule inhibitor, STM2457, significantly impeded tumor growth in LIHC cell lines, spheroids, and xenograft tumor model. Further, transcriptome and m^6^A sequencing of xenograft bodies unveiled that inhibition of METTL3-m^6^A altered genes enriched in SMAD and MAPK signaling pathways that are critical for tumorigenesis. These findings suggest that targeting METTL3 represents a promising therapeutic strategy for LIHC.

**Supplementary Information:**

The online version contains supplementary material available at 10.1186/s12935-023-03096-1.

## Introduction

N^6^-methyladenosine (m^6^A) is a prevalent and dynamic RNA modification found on eukaryotic mRNAs, playing a crucial role in post-transcriptional gene regulation. It is the most abundant internal modification on mRNA, occurring at the consensus motif RRm^6^ACH motif (R = G or A; H = A, C, or U) across thousands of coding and non-coding RNAs [1, 2]. The catalysis of m^6^A modification is carried out by a multicomponent methyltransferase complex (MTC), also known as “writers”, which includes key components such as METTL3, METTL14, Wilms’ tumor 1-associating protein (WTAP), Vir Like M6A Methyltransferase Associated (VIRMA), RNA Binding Motif Protein 15 (RBM15), and Zinc Finger CCCH-Type Containing 13 (ZC3H13) [3–5]. On the other hand, “erasers” such as fat mass and obesity-associated protein (FTO) and alkylated DNA repair protein AlkB homolog 5 (ALKBH5) are responsible for removing the m^6^A modification [6–8]. Additionally, specific RNA binding proteins including YTH Domain Containing Protein 1 (YTHDC1), YTH Domain Family, Member 1/2/3 (YTHDF1/2/3), and Insulin-Like Growth Factor 2 mRNA Binding Protein 1/2/3 (IGF2BP1/2/3) act as “readers” by recognizing m^6^A and subsequently regulating RNA stability, transport, translational initiation, and efficiency [9–15].

Methyltransferase-like 3 (METTL3), consisting of an S-adenosyl methionine-binding motif (MT-A70) and a zinc finger domain (ZFD), serves as a pivotal component of the m^6^A methyltransferase complex [3, 16]. Studies using stage-specific and germ cell-specific METTL3-knockout mouse models have demonstrated the essential role of METTL3 in various biological processes, including liver regeneration, hematopoietic stem cell differentiation, and meiosis initiation [17, 18]. Importantly, METTL3 has been implicated in human malignancy, acting as either an oncogene or a tumor suppressor [19].

In this study, we originally investigated the role of METTL3 in various cancers. We comprehensively analyzed the RNA and protein expression of METTL3, evaluated its prognostic significance, examined promoter methylation levels, assessed the impact on immune responses, and elucidated the cellular pathway networks associated with METTL3. The evaluations suggested METTL3 as a prognostic marker and a potential therapeutic target in LICH. Therefore, we subsequently focused our investigation on the oncogenic role of METTL3 in LIHC and aimed to explore the therapeutic potential by targeting METTL3 using a specific inhibitor, STM2457. To this end, we conducted experiments using LIHC cell lines, spheroids, and CDX models to assess the effects of METTL3 inhibition on tumor growth and progression.

Furthermore, to gain insights into the molecular mechanisms underlying the therapeutic effects of METTL3 inhibition, transcriptome-m^6^A sequencing and integrated analyses were performed. The analyses enabled us to identify the critical pathways affected by METTL3 inhibition in LIHC, including pathways related to cell cycle, metabolism, tumor survival and metastasis. By conducting this comprehensive study, we provided a deeper understanding of the role of METTL3 in cancer and its potential as a therapeutic target. The findings from our investigation have the potential to contribute to the development of novel treatment strategies for LIHC.

## Materials and methods

### Databases analyses

UCSC genome browser on human (http://genome.ucsc.edu/), National Center for Biotechnology Information (NCBI, https://www.ncbi.nlm.nih.gov), Human Protein Atlas (HPA, https://www.proteinatlas.org), Gene Expression Profiling Interactive Analysis (GEPIA, http://gepia2.cancer-pku.cn), Tumor Immune Estimation Resource (TIMER, http://timer.cistrome.org) portal, Somatic Mutations In Cancer (COSMIC), Clinical Proteomic Tumor Analysis Consortium (CPTAC, https://gdc.cancer.gov), and SangerBox (http://SangerBox.com/Tool) were used to analyze the relationship between METTL3 expression and tumorigenesis, gene methylation, immunity, and survival from a pan-cancer perspective [29–32].

By applying UALCAN portal (http://ualcan.path.uab.edu/analysis-prot.html) and MEXPRESS website (https://mexpress.be/), we examined the difference in METTL3 DNA promotor methylation levels between tumor and normal tissues [33].

TIMER2 (https://timer.cistrome.org) were used to explore the association between METTL3 expression and T cell regulatory and macrophage infiltration. Spearman’s rank correlation test was performed, and the P-value and partial correlation (cor) value were generated.

The LinkedOmics database (http://www.linkedomics.org/login.php) was applied to analyze genes that co-expressed with METTL3 in the LIHC cohort (TCGA_LIHC) [34].

### Clinical tissue samples and cell culture

All clinical tissues were collected from patients with primary LIHC who underwent surgery at the Jining First People’s Hospital, Shandong First Medical University. The samples were quickly stored in liquid nitrogen. According to the World Health Organization (WHO) criteria, hepatobiliary pathologists verified the pathological grade of each tissue sample. These specimens included 10 cases at grade I, 4 cases at grade II, and 2 cases at grade III. This study was approved by the institutional review boards of the hospitals, and written informed consent was obtained from all patients.

Huh-7 cell line was purchased from CLS (300,156), and cell line of HepG2, Hep3B, PLC/PRF5 and BEL-7404 were obtained from ATCC (HB-8065). All these cell lines were cultivated in Dulbecco’s modified Eagle’s medium (DMEM) supplemented with 10% FBS (Thermo Fisher Scientific, USA) and 5 ml of penicillin/streptomycin solution (P/S) (Cat. #0503, Scien Cell, USA). Hep3B, PLC/PRF5, BEL-7404 were obtained from American Type Culture Collection (ATCC, Manassas, VA, USA). Human normal liver cell line HepaRG^™^ was obtained from Johannes Kepler University Linz, Austria. LIHC cell lines were maintained in HepaRG^™^ Serum-free Induction Medium Supplement (5X) (HPRG750, Thermo Fisher Scientific, USA) supplemented with GlutaMAX^™^ (35050061, ThermoFisher). All cell lines were grown in a humidified incubator with 5% CO_2_ at 37 °C.

### Quantitative polymerase chain reaction (qPCR)

Total RNA was extracted from LIHC tumors/adjacent normal tissues and cell lines with FastPure^®^ Cell/Tissue Total RNA Isolation Kit V2 (RC112, Vazyme) according to the manufacturer’s instructions. For cDNA synthesis, 500 ng total RNA was used for reverse transcription in a 20 µl reaction volume with HiScript^®^III SuperMix for qPCR (R323, Vazyme). Then quantitative PCR (qPCR) was performed with ChamQ Universal SYBR green qPCR Master Mix (Q711, Vazyme) in an AB 7500 Fast Real-Time PCR system (Applied Biosystem). A t-test was used for statistical analyses, and P < 0.05 was considered statistically significant. GAPDH was used as endogenous control and each reaction was run in triplicate. The relative standard curve method was used to analyze the data by the 2−^ΔΔCt^ method and normalized to GAPDH.

Following primer sequences were used: GAPDH Forward: 5′-GAAAGCCTGCCGGTGACTAA-3′,Reverse: 5′-TTCCCGTTCTCAGCCTTGAC-3′; METTL3 Forward: 5′-ATCCCCAAGGCTTCAACCAG-3′,Reverse: 5′-GCGAGTGCCAGGAGATAGTC-3′; EGFR Forward: 5′-CCTACGGGCCAGGAAATGAG-3′, Reverse: 5′-ACCCAGCTGAAACTCTGACG-3′; SMAD3 Forward: 5′-GCACCCTGTCCAGTCTAACC-3′, Reverse: 5′-AGGCAGCACCCATAACTGAC-3′; DUSP5 Forward: 5′-TCGCTCAACGTCAACCTCAA-3′, Reverse: 5′-CTCTCCTCTCGCAGCTTCTG-3′; SPRY2 Forward: 5′-CATTCGCTCATCTGCCAGGA-3′, Reverse: 5′-GGTGTTTCGGATGGCTCTGA-3′.

### Immunoblot

Equal amount of prepared protein samples were loaded to an 10% SDS-PAGE gels for electrophoresis and then electrotransferred to a polyvinylidene difluoride membrane. The membrane was blocked with 5% BSA and incubated with primary antibodies overnight at 4 °C. After wash three times with 1 × TBST, the membrane was incubated with corresponding secondary antibody for 1 h and then detected with an ECL system. SPRY2 polyclonal antibody (Proteintech Cat # 11383–1-AP), EGF Receptor (D38B1) XP^®^ Rabbit mAb #4267 (Cell Signaling Technology, CST), p44/42 MAPK (Erk1/2) (137F5) Rabbit mAb #4695 (CST), Phospho-p44/42 MAPK (Erk1/2) (Thr202/Tyr204) (D13.14.4E) XP^®^ Rabbit mAb #4370 (CST) were applied in immunoblot.

### Flow cytometry

Flow cytometry (BD Biosciences, Franklin Lakes, NJ, USA) was used to analyze apoptosis and cell cycle. Briefly, apoptosis was evaluated using an Annexin V-APUSAC 7AAD apoptosis detection kit (MultiSciences Biotech, Westlake Village). Cells were trypsinized, rinsed twice with PBS, and resuspended in the Annexin V Binding Buffer at a concentration of 1 × 10^6^ cells/ml. Following transferring 100 µl of cell suspension into a test tube, 5 µl of AnnexinV conjugate and 5 µl of 7-AAD solution were added to each sample for 15 min at room temperature in the darkness. Finally, 400 µl of Annexin V binding buffer was added and analyzed using flow cytometry. The cell cycle was evaluated using a Cell Cycle Staining Kit (MultiSciences, Hang Zhou, China). Cells were trypsinized and washed with PBS twice. Each sample was incubated with a DNA staining solution containing 1 ml and 10 µl RNase for 30 min at room temperature in the dark and then subjected to analysis using flow cytometry.

### TUNEL assay

Cells were washed with PBS, fixed with 4% Paraformaldehyde, and then incubated with 50 µl of TUNEL reaction solution containing terminal deoxynucleotidyl transferase (TdT) and Cyanine3-labeled dUTP in the wet box at 37 °C for 20 min. After washing with PBS, the cells were observed and photographed under a microscope at 570 nm. Then the number of apoptotic cells was counted by ImageJ 1.49 V software.

### EdU assay

The Huh7 cells were seeded in a 6-well chambered cover glass (1 × 10^5^/well). After being cultured for 24 h, the cover glass was incubated with EdU working solution (100 nM) for 2 h. Cells were washed, fixed, permeabilized, and stained according to the instructions. After incubation with 300 µl hoechst33342 in the dark for 30 min, the cover glasses were visualized under a fluorescence microscope.

### Spheroid preparation and METTL3 inhibition with STM2457

24-well plate was pre-coated with 500 µl of 20 mg/ml PolyHEMA solution (dissolved in EtOH) (Sigma, P3932), and then was air-dried at 37 °C overnight. Huh7 or HepG2 cells were seeded as 1 × 10^4^ cells/well in DMEM medium containing 1 mg/ml Cultrex matrix (R&D systems, 3445–005-01) and cultured for 5 days to allow spheroid formation. A serial dilution of STM2457 (DMSO solvent control, 1 µM, 10 µM, or 50 µM) was then added into the medium to treat spheroids for 48 h. Spheroid number and size were counted and measured using GIMP software. To determine cell viability, ghost dye was added to the spheroid culture. After 20 min of incubation, spheroids were harvested, washed with PBS, and dissociated into a single cell population with Trypsin–EDTA. Cell viability was determined by flow cytometry.

### Cell-derived xenograft tumor growth

Huh7 cells were inoculated subcutaneously into Balb/c nude mice (1 × 10^7^ cells/mouse Pengyue, Shandong, China). The mice were randomly divided into treatment and control groups and were weighed weekly. STM2457 was diluted in a 20% 2-hydroxypropyl cyclodextrin vehicle. After 2 weeks of drug intervention with a dosage of 50 mg/kg, the subcutaneous tumor was dissected and photographed. Small hairpin RNA (shRNA) stably knocking down METTL3 were established by lentiviral infection in Huh7 cells, with the targeting sequence GCAAGAATTCTGTGACTATGG or scramble sequence.

### RNA sequencing and profiling

Total RNA was isolated from subcutaneously planted cell-derived xenograft tumors that were treated with STM2457 or vehicle using the enrichment method. Briefly, magnetic beads carrying Oligo dT primers were used to obtain mRNA with poly-A tails. The mRNA was subsequently fragmented into 130–160 nucleotide units. Quality assessment was performed with a Nanodrop ND-1000 Spectrophotometer (NanoDrop Technologies) and Agilent 2100 Bioanalyzer (Agilent). Reverse transcription was performed with random hexamers, and the transcription products were used to synthesize double-stranded cDNA. After purification and recovery, sticky-end ligation and fragment size selection, PCR amplification was performed, and the constructed library was sequenced. The sequencing data were imported into the quality control (QC) and filtering software SOAP-nuke, which was developed by BGI. Sequencing were perfoming by using the DNBSEQ platform. After obtaining clean data, HISAT was used to align the sequencing data to the reference genome sequence. After the second QC step, gene quantification and expression levels were quantified.

### m^6^A immunoprecipitation sequencing and profiling

The m^6^A immunoprecipitation (MeRIP) sequencing service was provided by CloudSeq Inc. (Shanghai, China). Total RNA was subjected to immunoprecipitation with the GenSeq m^6^A-IP Kit (GenSeq Inc.) by following the manufacturer’s instructions. Briefly, RNA was randomly fragmented to ~ 200 nt by RNA Fragmentation Reagents. Protein A/G beads were coupled to the m^6^A antibody by rotating at room temperature for 1 h. The RNA fragments were incubated with the bead-linked antibodies and rotated at 4 °C for 4 h. After incubation, the RNA/antibody complexes were washed for several times, and then, captured RNA was eluted from the complexes and purified. RNA libraries for IP and input samples were then constructed with GenSeq Low Input Whole RNA Library Prep Kit (GenSeq, Inc.) by following the manufacturer’s instructions. Libraries were qualified using Agilent 2100 bioanalyzer and then sequenced in a NovaSeq platform (Illumina). We employed the MeRIP analysis pipeline developed by Chuan He's lab, as detailed in the manuscript published [35]. Subsequent bioinformatics analysis, which included the utilization of the RADAR R package, allowed for the identification and quantification of m^6^A sites.

### Statistical analysis

The HR and P-value were used to evaluate the significance of differences in survival. Pearson’s correlation coefficient and statistical significance were employed to assess the associations of gene expression, and the absolute value was utilized to determine the strength of the correlation. The results were regarded as statistically significance at *P < 0.05, **P < 0.01 and ***P < 0.001.

## Results

### The expression level of METTL3 in human cancers and association with prognosis

**Fig. 1:**
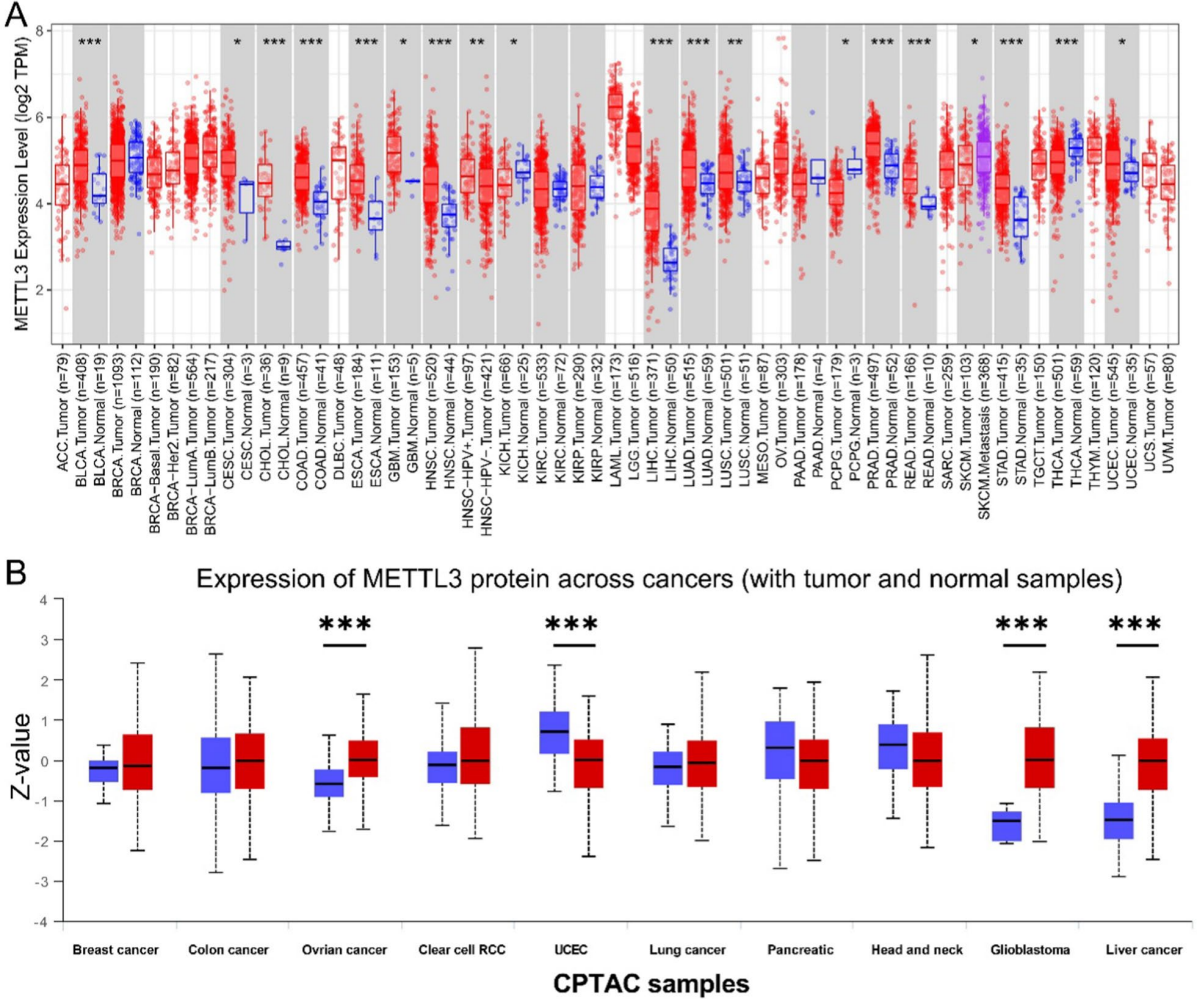
Characteristics and expression of METTL3 in normal tissues and cancers. **A** METTL3 mRNA expression in different cancers according to TIMER. **B** METTL3 protein expression data in 10 normal and tumor tissues (***P < 0.001)

To assess the expression level of METTL3 in various human cancers, we utilized the TIMER2 database for analysis across multiple TCGA cancer and normal tissues. Figure 1A depicted a significant upregulation of METTL3 expression in a variety of cancer types (see Abbreviation), when compared to their respective normal tissues. Furthermore, an examination of the CPTAC dataset revealed enhanced expression levels of in liver cancer, glioblastoma, and ovarian cancer, while decreased levels were observed in UCEC, pancreatic cancer, and head and neck cancer (Fig. 1B).

**Fig. 2:**
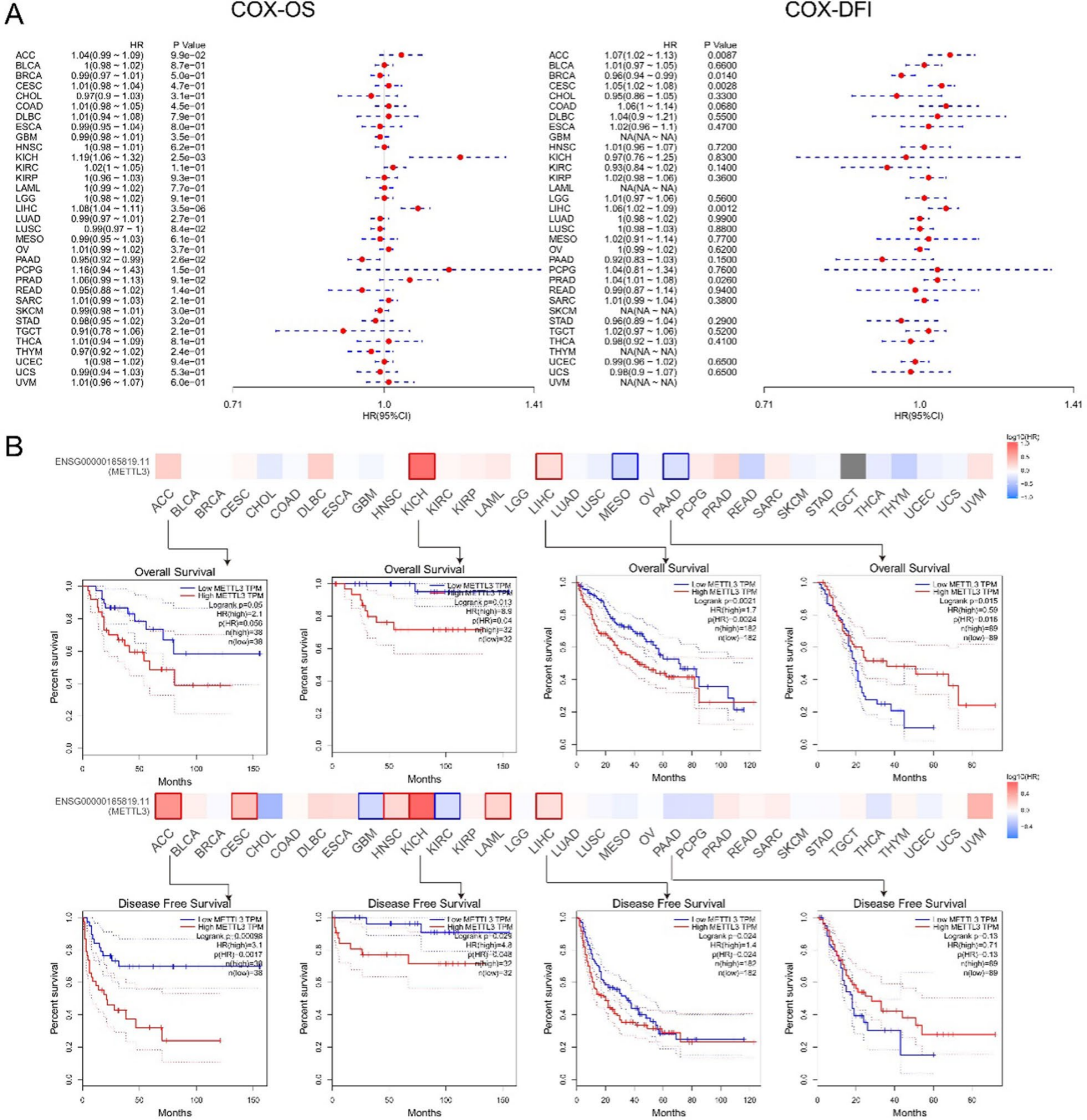
Correlation between METTL3 mRNA expression and prognosis of cancers. **A**The relationships between METTL3 expression and OS and DFI prognosis of different cancers in the “Gene-KM plotter” module of SangerBox. **B** Correlation between METTL3 gene expression and survival prognosis of cancers in TCGA. We used the GEPIA2 tool to perform overall survival and disease-free survival analyses of different tumors in TCGA by METTL3 gene expression. The survival map and Kaplan–Meier curves with positive results are given

Cox analysis revealed that METTL3 expression served as a significant hazard factor influencing overall survival (OS) and disease-free interval (DFI) in KICH, LIHC, PRAD, and PCPG (Fig. 2A). Moreover, GEPIA2 database demonstrated that METTL3 overexpression was associated with poor OS in the patients with ACC, KICH, LIHC, UVM, DLBC, PRAD, or LAML in TCGA cohorts. Conversely, downregulated METTL3 expression was correlated with poor OS in patients with MESO, PAAD, READ, THYM, LUSC, and CHOL (Fig. 2B**,** upper panel). A similar trend was found for DFS, with high METTL3 expression positively associated with poor DFS in patients with ACC, KICH, LIHC, CESC, HNSC, KIRC, LAML, and UVM, while negatively correlated with poor DFS in patients with CHOL, GBM, KIRC, PAAD, LUSC, and PCPG (Fig. 2B, lower panel).

### Analysis of METTL3 promoter methylation and gene alteration in cancers

We then analyze the methylation level of the METTL3 promoter using the “TCGA analysis methylation” function of UALCAN [36–38]. Our findings, as depicted in Additional file 1: Figure S1A, revealed a significant reduction in the methylation levels of the METTL3 promoter in LIHC, LUAD, BLCA, BRCA, HNSC, PRAD, THCA, SARC, and UCEC compared to their adjacent normal tissues. Conversely, an increase in promoter methylation was observed in certain other cancer types. To further explore the association between METTL3 promoter DNA methylation and pathogenesis in LIHC, we analyzed the TCGA cohort using the MEXPRESS approach. We observed a negative correlation between DNA methylation at several probes within the METTL3 non-promoter region, such as cg08233448 (P < 0.001, R = − 0.185) and cg02766594 (P < 0.001, R = − 0.221), and its gene expression (Additional file 1: Figure S1B). Similar results were observed in other cancer types, including ACC and LUAD. These analyses support the notion that overexpression of METTL3 in LIHC and other tumor types could be attributed to hypomethylation of its promoter region.

### METTL3 expression is related to immunity

The presence of tumor-infiltrating immune cells plays a crucial role in cancer progression, metastasis, and treatment outcomes by modulating the tumor microenvironment [39–41]. To understand the association of METTL3 expression and immune cell infiltration in cancers, we utilized the TIMER database. Our analysis, as shown in Additional file 2: Figure S2A, revealed significant associations between METTL3 expression and diverse immune cell infiltrates in KIRC, LIHC, PAAD, PCPG, and SKCM. For instance, in LIHC, high METTL3 expression correlated with increased immune cell infiltration (Additional file 2: Figure S2A) and poor prognosis (Additional file 2: Fig. 2B). Positive correlations were observed between METTL3 expression and the levels of infiltrating CD8 + T cells (r = 0.143, P = 7.87e-03), CD4 + T cells (r = 0.39, P = 5.73e-14), macrophages (r = 0.356, P = 1.25e-11), neutrophils (r = 0.304, P = 8.7e-9), and DCs (r = 0.309, P = 6.21e-9) in LIHC. Moreover, we employed TIMER2 to examine the potential correlations between METTL3 expression and the infiltration levels of individual immune cell types across various cancers in TCGA. Notably, METTL3 expression exhibited positive associations with macrophage infiltration in UVM, LIHC, and DLBC, while negative correlations were observed in UCEC, SARC, LGG, KRP KIRC, GBM, CESC, and BRCA-LumA (Additional file 2: Figure S2B). Regarding regulatory T cell (Tregs) infiltration level, METTL3 expression showed positive correlations with UVM, LIHC, LUAD and BRCA, but they were negative correlated in THCA and HNSC in most algorithms (Additional file 2: Figure S2B). Scatter plots presenting the positive correlation between METTL3 expression level and infiltrate estimation values of macrophages and Tregs in LIHC, based on TIMER algorithms, were also provided in Additional file 2: Figure S2C. These findings highlight the distinct patterns of correlations between METTL3 expression and immune cell infiltration in LIHC [42–47].

### Analysis of the METTL3-related genes: biological functions and signaling pathways

To gain insights of the biological functions and signaling pathways associated with METTL3, we conducted an analysis using the LinkedOmics database and focused on LIHC [34]. The heatmap in Additional file 3: Figure S3A, B represents the top 50 genes that were positively and negatively correlated with METTL3 expression. The analysis suggested that positively associated genes are enriched in cell cycle, DNA replication, and stemness-related pathways such as WNT, NOTCH signaling pathways.

Next, we utilized the Gene Set Enrichment Analysis (GSEA) function module to determine the major Kyoto Encyclopedia of Genes and Genomes (KEGG) pathways and Biological Process categories of Gene Ontology (GO) terms for METTL3 co-expressed genes. The KEGG pathway analysis of METTL3-associated genes revealed several pathways that were enriched in LIHC, including the mRNA surveillance pathway, RNA transport, cell cycle, oocyte meiosis, DNA replication, ribosome biogenesis, RNA degradation, WNT signaling pathway, and phosphatidylinositol signaling cascade (Additional file 3: Figure S3C). The GO term annotation (GO_BP) indicated that METTL3-related genes primarily participated in processes such as ribonucleoprotein complex localization, RNA localization, DNA-templated transcription, termination, regulation of gene expression, epigenetics, mRNA processing, cell cycle G2/M phase transition, DNA replication, and others (Additional file 3: Figure S3D). Additionally, METTL3 expression was negatively associated with oxidative phosphorylation, cell adhesion molecules, multiple metabolism/ homeostasis, and drug catabolism processes in LIHC (Additional file 3: Figure S3C and S3D).These findings provide valuable insights into the potential functions and signaling pathways associated with METTL3, shedding light on its role in tumorigenesis and highlighting potential targets for further investigation in LIHC.

### Experimental identification of METTL3 as a biomarker and therapeutic target in LIHC

**Fig. 3:**
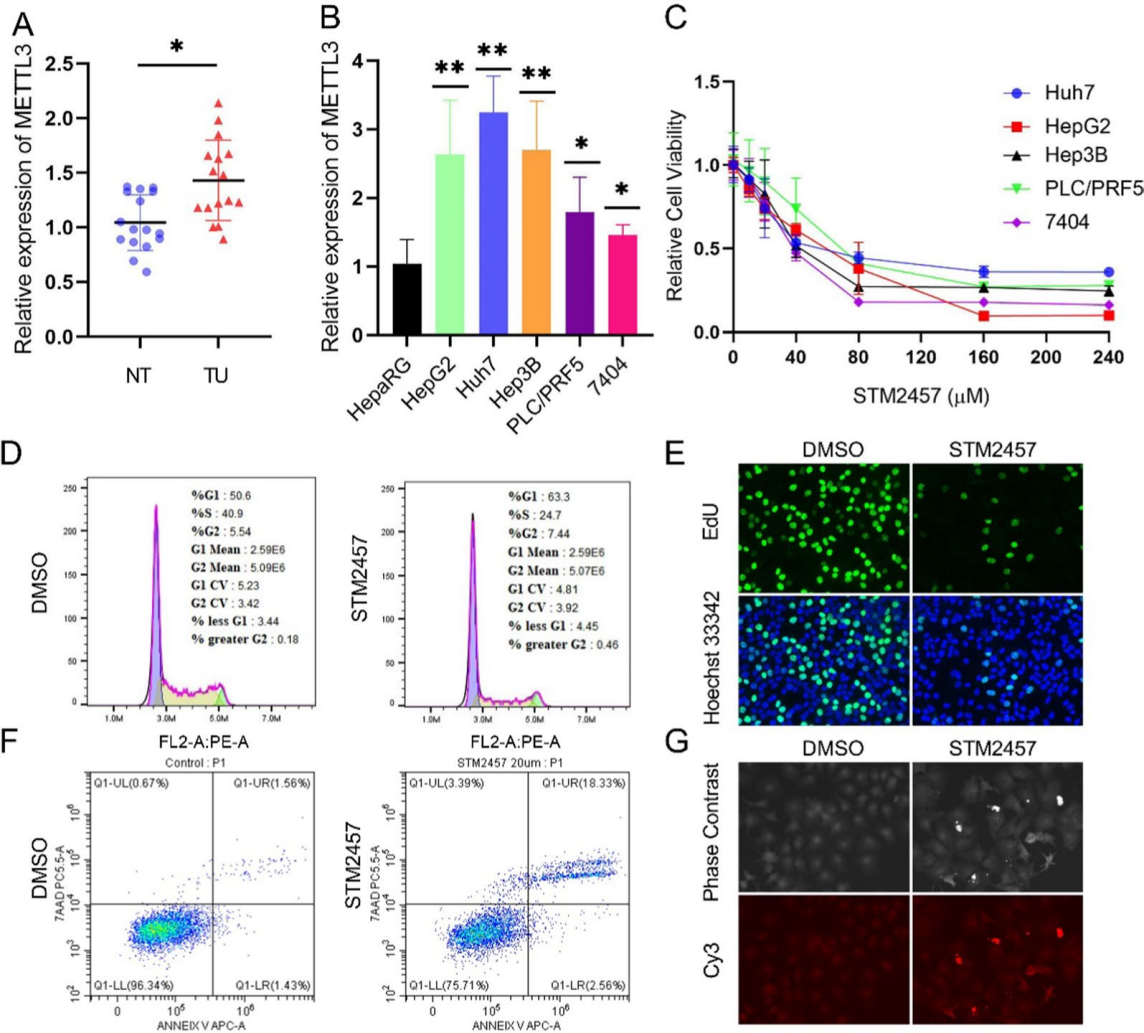
Targeting METTL3 in LIHC cell lines. **A** and **B** The expression of METTL3 in 16 pairs of LIHC tumor (TU)/normal tissue (NT) and LIHC cell lines. **C** Relative cell viability following treatment of LIHC cells with METTL3 inhibitor STM2457. **D** Flow cytometry of G1 phase cell, S phase cell, and G2 phase cell percentage after treatment of Huh7 cells with STM2457. **E** The EdU incorporation in Huh7 cells was suppressed by METTL3 inhibitor. **F** and **G** Flow cytometry and TUNEL assay indicated apoptotic cells in Huh7 cells after treatment with METTL3 inhibitor

Quantitative PCR analysis showed significantly higher expression of METTL3 in primary liver tumors compared to adjacent non-tumor tissues (n = 16) (Fig. 3A). Liver cancer cell lines, including HepG2, Huh7, Hep3B, PLC/PRF5, and BEL7404, also exhibited elevated METTL3 expression compared to the immortalized normal hepatocellular cells HepaRG (Fig. 3B).

To assess the therapeutic potential of targeting METTL3, liver cancer cell lines were treated with METTL3 inhibitor STM2457 at different concentrations [48]. After 24 h, the IC50 values of STM2457 were determined for each cell line and steady inhibition of cell viability were observed (Fig. 3C). Further flow cytometry analyses of the cell cycle revealed that inhibition of METTL3 with STM2457 led to a decrease in S-phase cells and an increase in G1-phase cells. DNA replication was obviously arrested upon STM2457 treatment, as shown by EdU incorporation (Fig. 3D and E). Flow cytometry and TUNEL assay also indicated an increase in apoptotic Huh7 cells upon METTL3 inhibition (Fig. 3F, G).

**Fig. 4:**
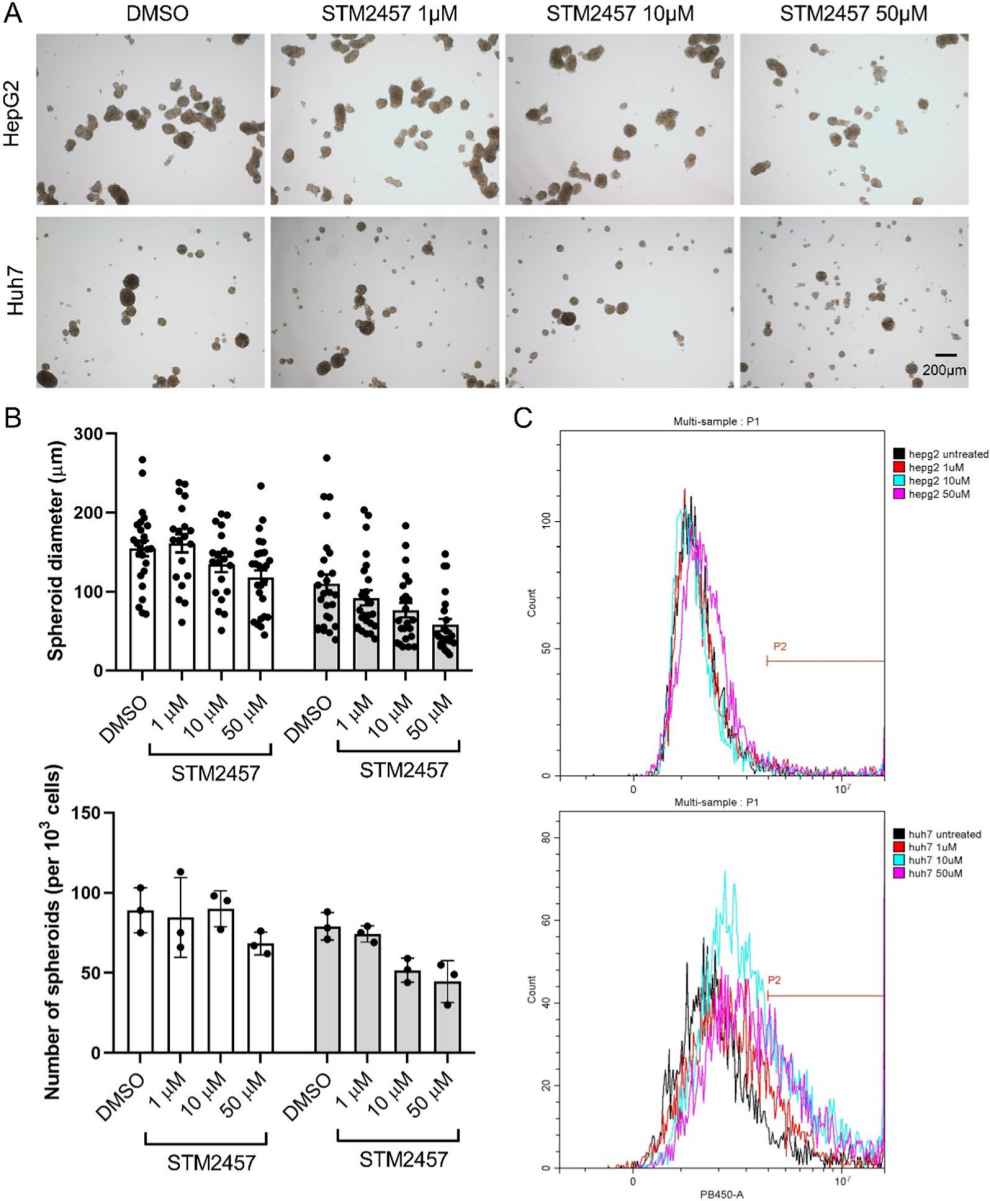
Targeting METTL3 in cell-derived spheroids. **A** METTL3 inhibitor attenuated the growth of Huh7 cell and HepG2 cell-derived spheroids in a dose-dependent manner. **B** The forming of spheroids was quantified by size and numbers; data from HepG2 cells is presented as blank bars and the data from Huh7 cells is presented as grey bars. **C** The viability of spheroids were quantified with ghost dye staining

Spheroids and mouse xenograft experiments were performed to validate the effects of METTL3 inhibition in LIHC tumorigenicity. STM2457 treatment resulted in a dose-dependent suppression of the number and the size of Huh7 and HepG2 spheroids (Fig. 4A, B). Cell viability measurements by ghost dye indicated that the inhibitory effects of STM2457 were, at least in part, due to the induction of cell death (Fig. 4C).

**Fig. 5:**
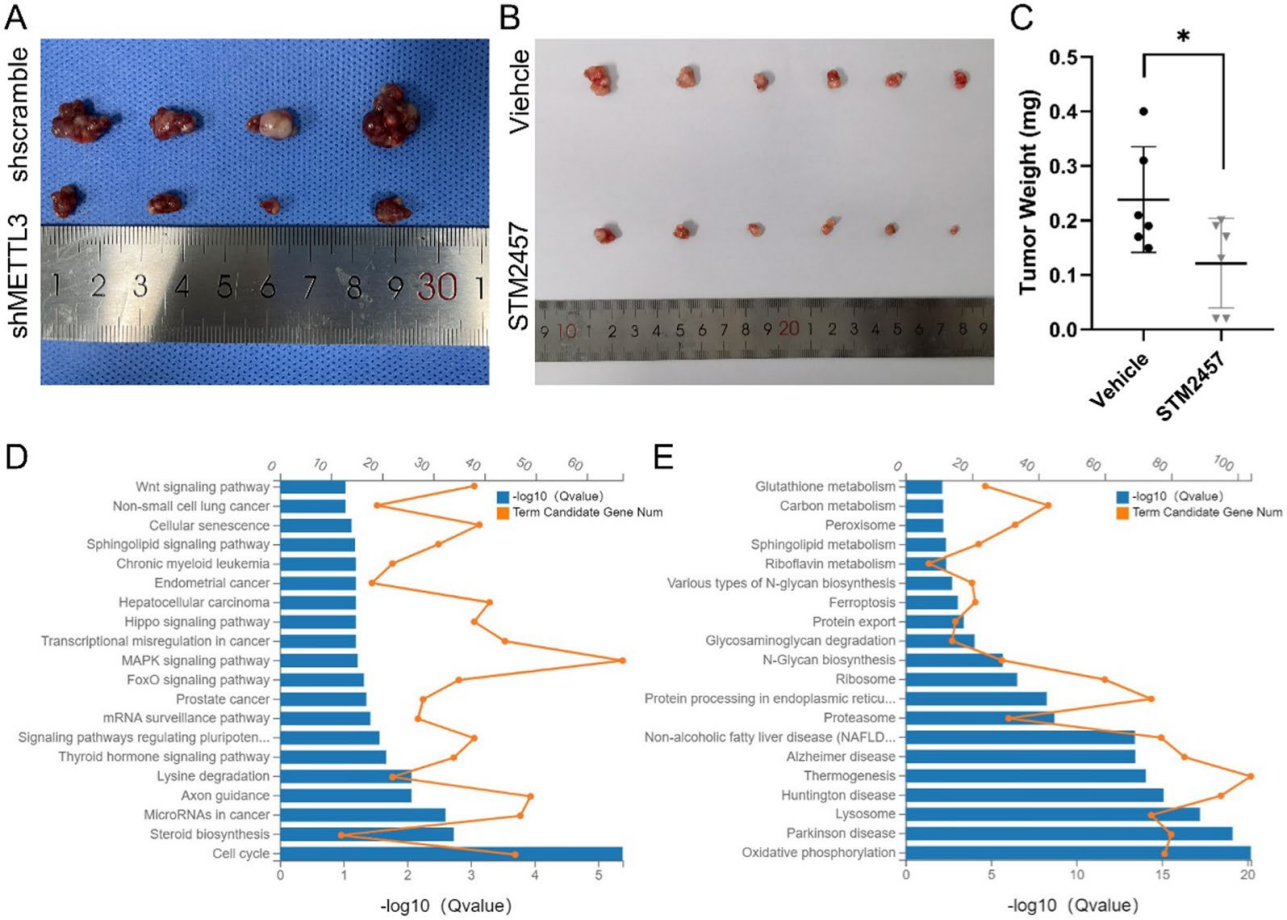
Inhibition of METTL3 suppressed CDX tumor growth in nude mice. **A**, **B** Knocking-down METTL3 and inhibition on METTL3 significantly suppressed tumorigenicity in the subcutaneously implanted mouse model. **C** Weight of the dissected tumors in vehicle group and STM2457 treatment group. **D** The top 20 most significantly enriched Kyoto Encyclopedia of Genes and Genomes (KEGG) pathways of downregulated and upregulated genes in CDX tumors treated with STM2457 when compared with vehicle. Data were shown as replicated experiments. Term candidate gene number and Q-value are indicated

Similar to previous report [21], knocking-down of METTL3 caused reduction of size in subcutaneously implanted tumors in nude mice (Fig. 5A). In vivo experiments also demonstrated that treatment with the METTL3 inhibitor significantly suppressed tumor growth (Fig. 5AC) [21]. Transcriptome profiling of the xenograft tumors treated with and without STM2457 demonstrated that the down-regulated genes were primarily enriched in the cell cycle pathway, MAPK signaling pathway, and multiple cancer-related pathways (Fig. 5D). Up-regulated genes upon METTL3 inhibition were mostly enriched in the oxidative phosphorylation pathway, lysosome and ferroptosis, to some extent (Fig. 5E).

**Fig. 6:**
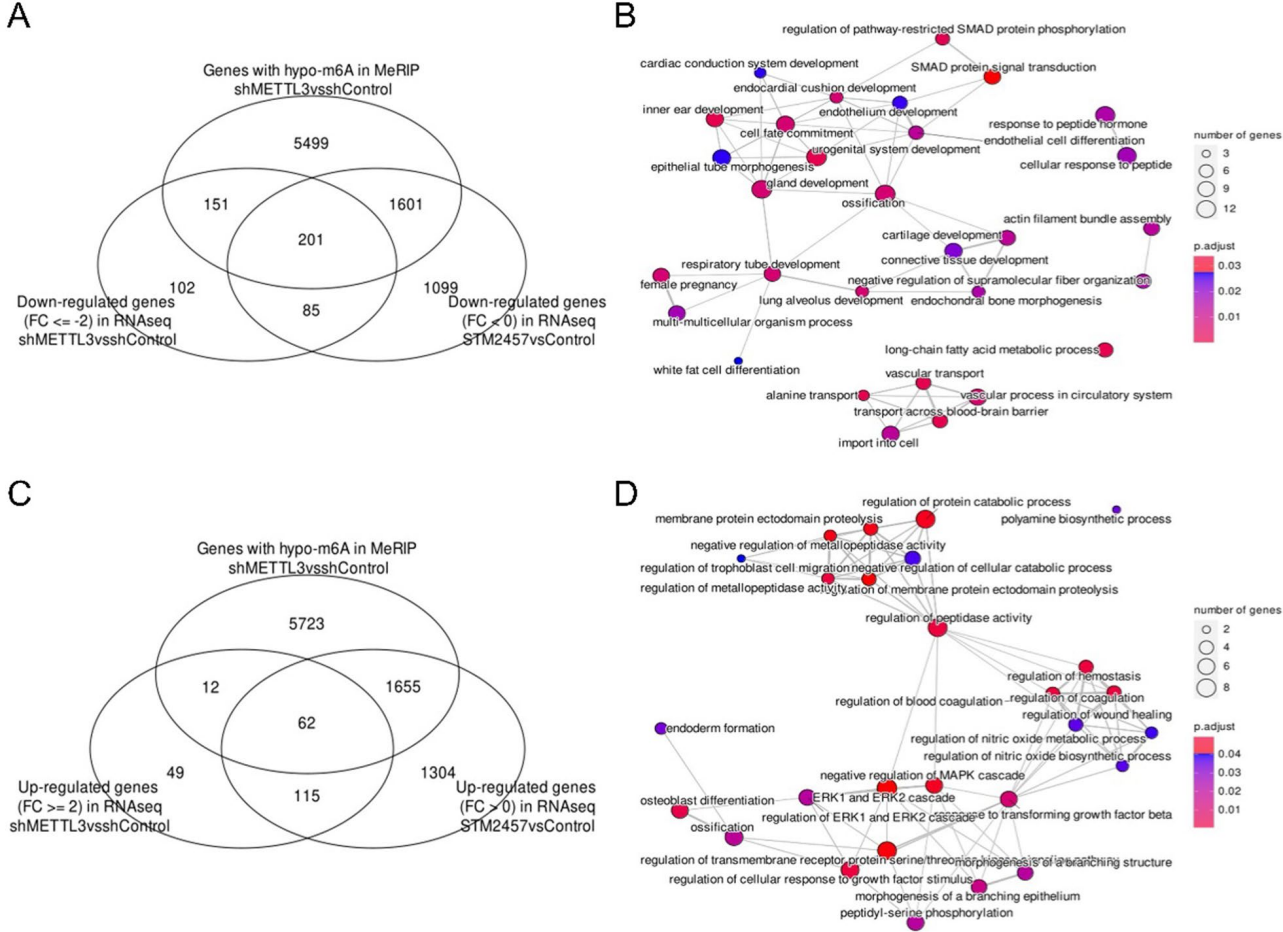
Integrated analyses of transcriptome and METTL3-m^6^A dependent pathways. **A** Venn diagram illustrating the intersection of genes identified as m^6^A-hypomethylated by MeRIP analysis in xenograft with shMETTL3_cells compared to shControl_cells, genes identified as down-regulated by RNA-seq analysis in shMETTL3_cells compared to shControl_cells, and genes identified as down-regulated by RNA-seq analysis in STM2457-treated xenograft bodies compared to control mice. **B** Enriched Gene Ontology Biological Process (GO_BP) terms for the overlapping genes identified in A. **C** Venn diagram illustrating the intersection of genes identified as m^6^A-hypomethylated by MeRIP analysis in xenograft bodies with shMETTL3_cells compared to shControl_cells, genes identified as up-regulated by RNA-seq analysis in shMETTL3_cells compared to shControl_cells, and genes identified as up-regulated by RNA-seq analysis in STM2457-treated nude mice compared to control mice. **D** GO_BP terms for the overlapping genes identified in **C**.

**Fig. 7:**
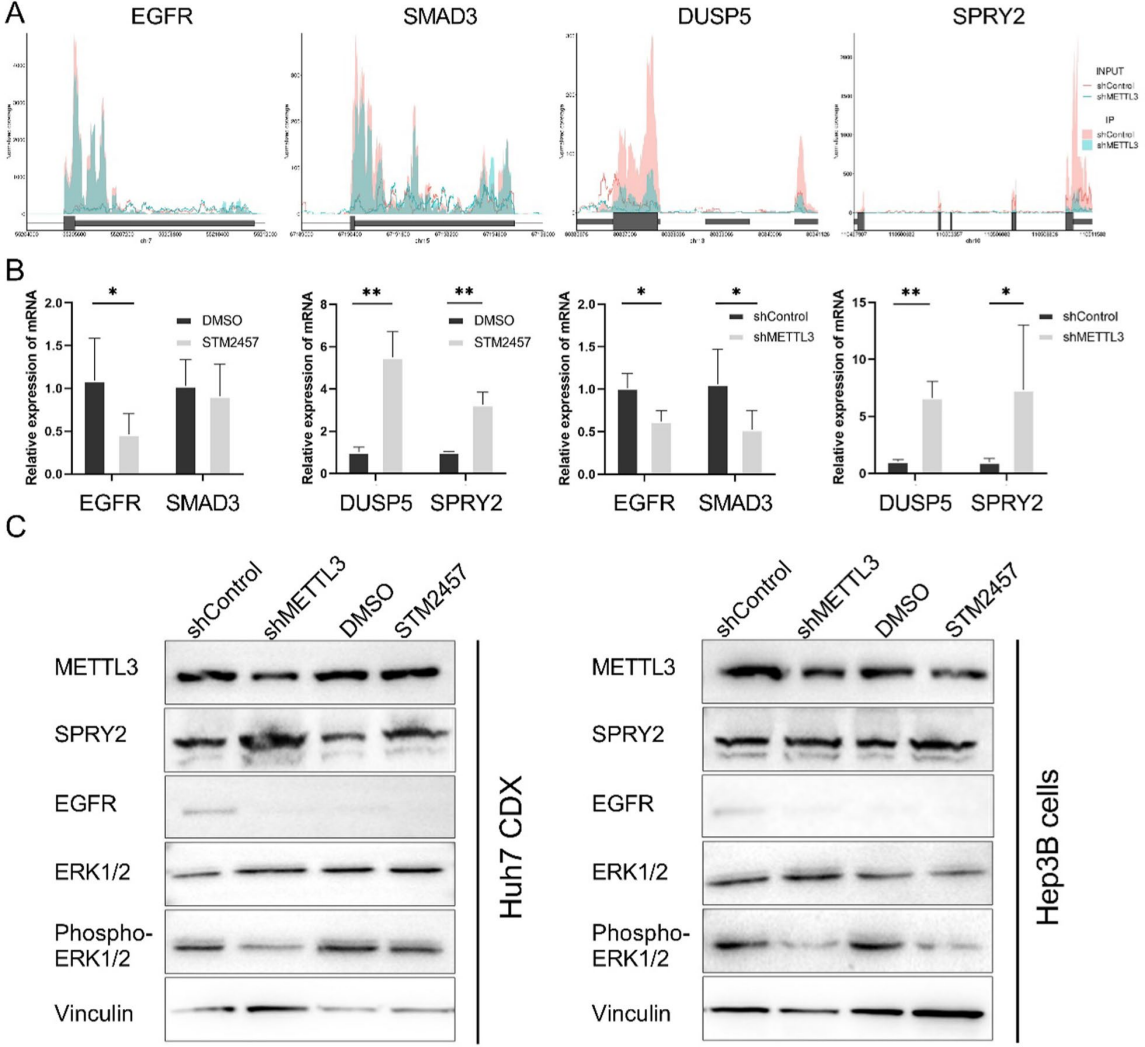
Targeting METTL3 reverses activation of MAPK cascades. **A** Overlay m^6^A peaks revealed SMAD3 and MAPK pathway-related genes as METTL3 targets. **B** qRT-PCR verification of the expression of EGFR, SMAD3, DUSP5, and SPRY2 genes in control Huh7 xenografts and that treated with STM2457 or with METTL3 knocking-down. **C** Immunoblot shows expression of METTL3, SPRY2, EGFR, ERK1/2, and phosphorylated ERK1/2 in control Huh7 xenografts or Hep3B cells and their counterparts treated with STM2457 or with METTL3 knocking-down.

Through integrated analyses of transcriptome and m^6^A sequencing data obtained from xenograft bodies, our objective was to identify the specific carcinogenic pathways that were directly regulated in an METTL3-m^6^A-depended manner in LIHC. We compared the down-regulated genes and up-regulated genes with significantly altered m^6^A levels in the control group to those in which METTL3 was knocked down. To ensure the fidelity of the results, the identified METTL3-m^6^A regulated genes were intersected with the genes that were changed upon treatment with STM2457 (Fig. 6A, C). Subsequently, the overlapped gene sets were analyses with GO term annotation. The analyses demonstrated that long chain fatty acid metabolic process, small mother against decapentaplegic (SMAD) protein phosphorylation and SMAD protein signaling transduction (such as SMAD3, SMAD4, SMAD5 and SMAD9) were greatly sustained by METTL3-m^6^A, while genes that involved in negative regulation of mitogen-activated protein kinase (MAPK) cascade, such as dual specificity phosphatase 5 (DUSP5) and sprouty homolog 2 (SPRY2), were dramatically enhanced when METTL3-m^6^A was inhibited (Fig. 6B, D). We also observed direct regulation of EGFR by METTL3-m^6^A. Using m^6^A peak density and qRT-PCR, the METTL3-m^6^A regulated genes SMAD3, EGFR, DUSP5, and SPRY2 were further assessed. In LIHC cancer cell Huh7 xenografts, knockdown of METTL3 or treatment with STM2457 resulted in decreased m^6^A modification and up-regulated expression of DUSP5 and SPRY2, suggesting that METTL3 suppression has a repression effect on the MAPK cascades (Fig. 7A, B) [49–51]. Immunoblotting confirmed that METTL3 suppression resulted in a significant reduction in phosphorylation of MAPK’s downstream targets, extracellular signal-regulated kinases 1 and 2 (ERK1/2) (Fig. 7C).

**Fig. 8:**
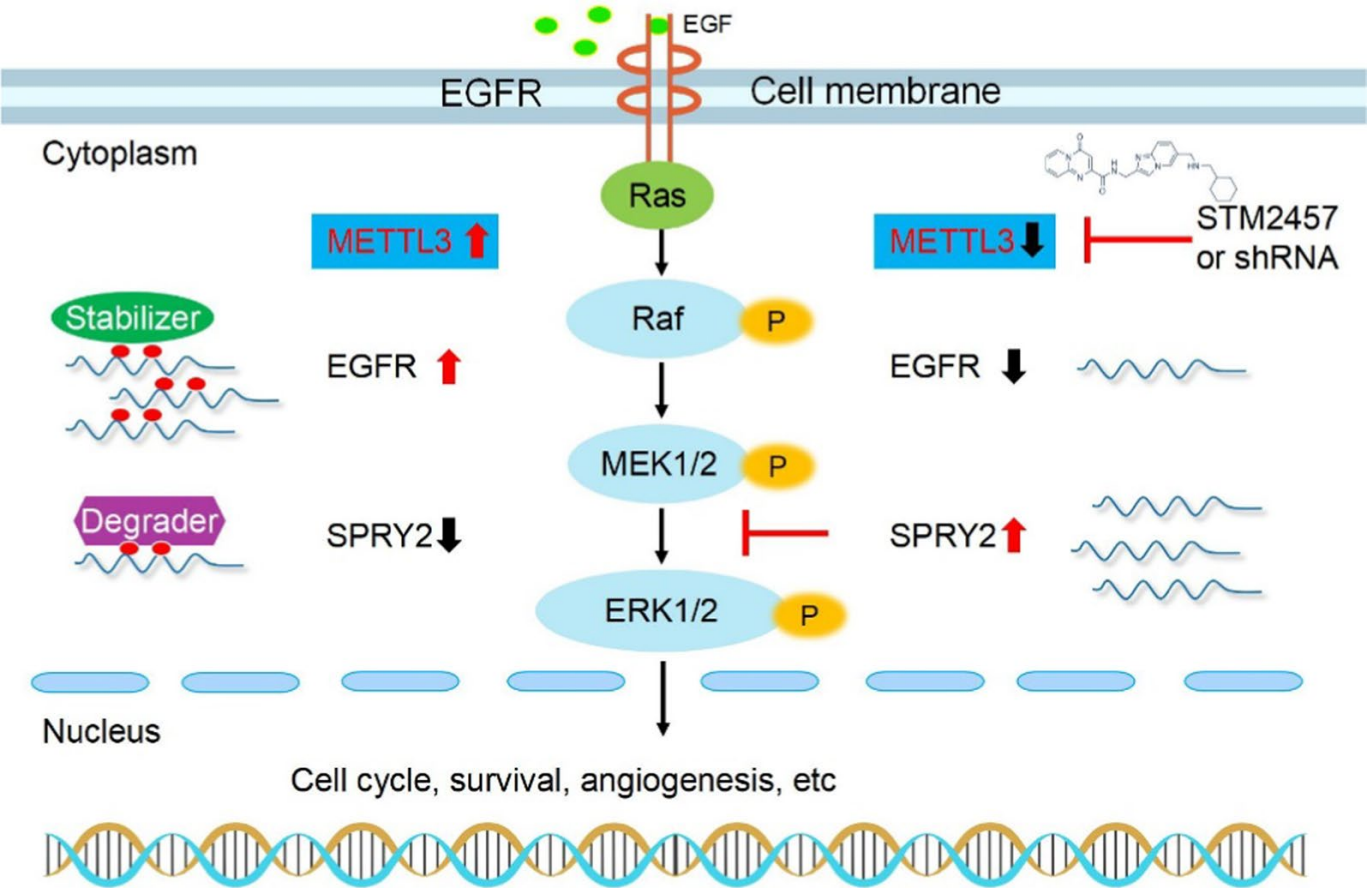
Working model of METTL3-mediated modulation of m^6^A-modified mRNAs within the MAPK cascades. mRNAs were methylated by the methyltransferase METTL3. The stability of EGFR, when modified by m^6^A, was observed to increase, while the degradation of SPRY2, when modified by m^6^A, was found to occur preferentially. The targeting of METTL3 leads to the stable translation and accumulation of SPRY2, which subsequently suppresses the MAPK cascades

These findings collectively suggest that METTL3 plays a pivotal role in promoting the growth of liver cancer cells primarily by affecting extracellular signaling pathways and cell cycle via modulating MAPK cascades, as illustrated in Fig. 8. The experimental data support the potential of METTL3 as a biomarker and therapeutic target in liver cancer.

## Discussion

METTL3 plays a pivotal role in various biological processes and is potentially associated with human diseases. Previous studies have implicated METTL3 as a potential therapeutic target in multiple cancer types by influencing RNA surveillance, and protein translation [21, 48, 52, 53]. In colorectal carcinoma (CRC), METTL3 promotes stemness and tumor progression by regulating the expression of SOX2 through an m^6^A-IGF2BP2-dependent mechanism [20]. In LIHC, METTL3 induces cell proliferation and migration by silencing cytokine signaling 2 (*SOCS2*) [21]. In acute myeloid leukemia (AML), METTL3 promotes leukemogenesis and inhibits myeloid differentiation, potentially through the targeting of oncogenic genes such as *MYC* and *CEBPA *[22, 23]. Moreover, upregulation of METTL3 has also been observed in various other cancer types, including glioblastoma, lung, stomach, bladder, pancreas, and prostate cancers [24]. Increasing evidence suggests that numerous m^6^A targets contribute to tumorigenesis, including *Snail*, *CTNNB1*, *NANOG*, *APC,* and genes associated with the Akt/mTOR pathway [11, 25–28] However, conflicting reports have indicated that METTL3 may function as a tumor suppressor in certain tumor types. Reduced expression of METTL3 has been observed in renal cell carcinoma, bladder cancer, and CRC [24]. Collectively, these studies provide substantial evidence the critical involvement of abnormal METTL3 expression and m^6^A modification in carcinogenesis. The comprehensive analysis conducted in this report evaluated the expression, prognostic value, methylation level, immunology, and signaling pathways associated with METTL3 in various cancer types. The overexpression of METTL3 was observed in most tumors compared to adjacent normal tissues, and this upregulation was linked to poor overall survival (OS) and disease-free interval (DFI) in several cancer types, particularly in LIHC. There was a negative correlation between promoter or gene methylation and METTL3 expression, indicating that aberrant methylation contributes to overexpression of METTL3 and oncogenesis in LIHC. Gene amplification was also suggested as a potential mechanism for METTL3 overexpression in certain cancers. Pathway analysis with LinkedOmics identified potential pathways including cell cycle and DNA replication were associated with METTL3 in LIHC carcinogenesis, further emphasizing its tumorigenic role and the pathways mediated by METTL3 [34]. Furthermore, the analysis revealed correlations between METTL3 expression and the expression of immune cell-related genes, such as CD8 + T cell, regulatory T cells, and macrophages, in various cancer types, particularly in LIHC. The immunology analyses also provided a new perspective for studies of the role of METTL3 in immunity modulation in LIHC. While the manuscript was being prepared, a number of research revealed that METTL3 may control PD-L1 expression or be involved in immunotherapy for lung cancer and colorectal cancer [54–56], implying the complicated role of METTL3 in carcinogenesis.

Significantly, experimental studies were conducted, confirming the high expression of METTL3 in liver cancer clinical tissues and cell lines. Inhibiting METTL3 using the specific inhibitor STM2457 demonstrated its ability to suppress cell growth by inhibiting DNA replication and inducing apoptosis. Additionally, STM2457 was able to reduce the renewal of LIHC cancer stem cells, as evidenced by spheroid culture. In vivo studies using xenograft models further demonstrated the inhibitory effects of STM2457 as well as knocking-down of METTL3 on tumor growth. The RNA-seq analysis revealed that genes exhibiting substantial changes were predominantly enriched in cell cycle and the MAPK signaling pathways, consistent with our analysis through LinkedOmics database (Fig. 5D and Additional file 3: S3C). This response to METTL3 inhibition, however, may have been indirectly mediated by upstream growth signals. Notably, our integrated profiling unveiled that METTL3-m^6^A played a direct role in regulating key extracellular signaling pathways involved in epithelial mesenchymal transition (EMT), tumor growth or metastasis, including the SMAD protein signaling transduction and negative regulation of MAPK pathways [57–60]. To enhance the confidence in the transcriptomic and methylome analyses, we further validated the key pathways and genes in tumor bodies using qRT-PCR and western blot. Upon inhibition of METTL3, the expression of DUSP5 and SPRY2 genes, which have been reported to oppose MAPK/ERK and cell proliferation, increased significantly. In contrast, a high level of SPRY2 protein is associated with a lower level of EGFR and phospho-ERK1/2. [21, 61] The collective findings of bioinformatic analysis and experimental investigations provide compelling evidence for the robust regulation of MAPK pathway by METTL3. Based on our transcriptomic-methylome analysis and subsequent experimental validation, it is postulated that METTL3 has the potential to induce cell cycle activation by inhibiting the suppressor molecules within the MAPK pathway.

## Conclusions

This study concludes with a comprehensive analysis of the expression, prognostic value, methylation level, immunology, and signaling pathways associated with METTL3. The findings shed light on the functional mechanism and therapeutic implications of METTL3 as a potential therapeutic target in LIHC. Validation of the critical signaling pathway and genes regulated by METTL3-dependent m6A modification and exploration of METTL3's complete role in tumorigenesis require additional study.

### Supplementary Information

**Additional file 1: Figure S1.** Methylation level of the METTL3 gene in different tumors and its association with gene expression. **A** Promoter methylation levels of the METTL3 in different cancer types compared to normal adjacent tissues. The Beta value indicates the level of DNA methylation ranging from 0 (unmethylated) to 1 (fully methylated). Different beta value cut-off has been considered to indicate hypermethylation [Beta value: 0.7 - 0.5] or hypo-methylation [Beta-value: 0.3 - 0.25]. **B** The MEXPRESS approach was used to analyze the DNA methylation level of the METTL3 with multiple probes. The promoter region probes were highlighted. The beta value of methylation, the Benjamini-Hochberg-adjusted P-value, and the Pearson correlation coefficients (R) are displayed.

**Additional file 2: Figure S2.** Correlation of METTL3 expression with immune infiltration level in diverse cancer types. **A** The correlation of the METTL3 expression with tumor purity and the abundance of immune cell infiltration. **B** The relationship between the expression of the METTL3 and the infiltration level of macrophages and Tregs across all cancer types in TCGA. TIMER algorithm analysis showed the potential correlation between the expression level of the METTL3 gene and the infiltration level of Tregs and macrophages in LIHC. **C** Scatter plots present the positive correlation between METTL3 expression level and infiltrate estimation value of macrophage and Treg cells in LIHC based on TIMER algorithms.

**Additional file 3: Figure S3.** Figure S3 METTL3 co-expressing genes in LIHC revealed by LinkedOmics. Heatmaps showing the top 50 genes positively (**A**) and negatively (**B**) correlated with METTL3 in LIHC. Red shows positively correlated genes and Blue represents negatively correlated genes. Significantly enriched KEGG pathways (**C**) and GO annotations(**D**) of METTL3 co-expressing genes in LIHC.

## Data Availability

The datasets used and/or analysed during the current study are available from the corresponding author upon reasonable request.

## Abbreviations

m^6^A: N6-methyladenosine
METTL3: Methyltransferase-like 3
MeRIP: M^6^A immunoprecipitation
SMAD: Small mother against decapentaplegic
MAPK: Mitogen-activated protein kinase
LIHC: Liver hepatocellular carcinoma
CHOL: Cholangiocarcinoma
STAD: Stomach adenocarcinoma
HNSC: Head and neck squamous cell carcinoma
ESCA: Esophageal carcinoma
DLBL: Diffuse Large B-cell lymphoma
BLCA: BLadder urothelial carcinoma
PRAD: PRostate ADenocarcinoma
READ: Rectum adenocarcinoma
LUAD: Lung adenocarcinoma
LUSC: Lung squamous cell carcinoma
KICH: Kidney chromophobe
DLBC: Lymphoid neoplasm diffuse large B-cell lymphoma
UCEC: Uterine corpus endometrial carcinoma
THYM: Thymoma
