# Supplementary figures and images for: Therapeutic effect and transcriptome-methylome characteristics of METTL3 inhibition in liver hepatocellular carcinoma
Source: Cancer Cell Int. 2023 Nov 27;23:298. doi: 10.1186/s12935-023-03096-1 (PMC10683134; doi:10.1186/s12935-023-03096-1)

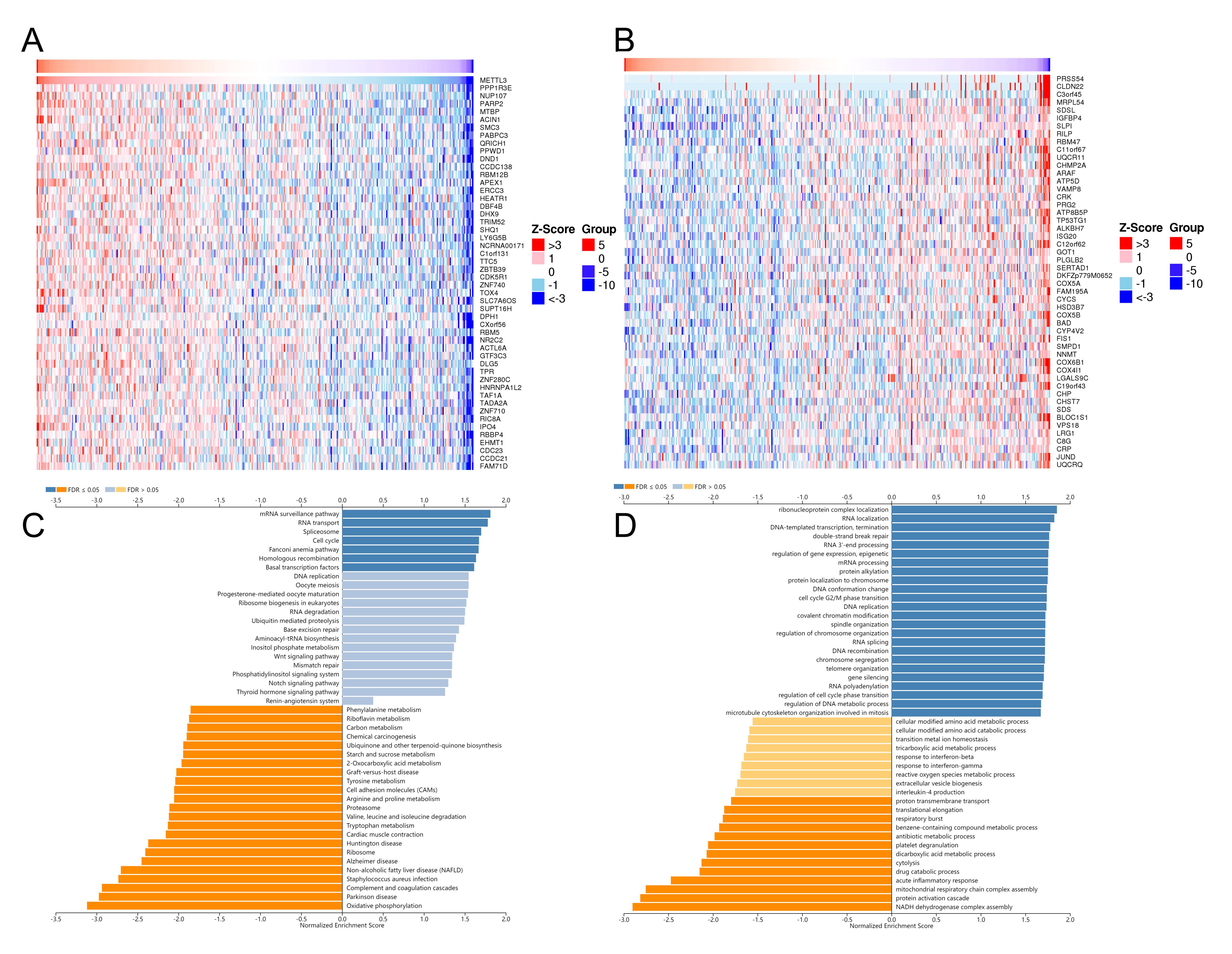

Supplement: Supplementary file 3 — Additional file 3: Figure S3. Figure S3 METTL3 co-expressing genes in LIHC revealed by LinkedOmics. Heatmaps showing the top 50 genes positively (A) and negatively (B) correlated with METTL3 in LIHC. Red shows positively correlated genes and Blue represents negatively correlated genes. Significantly enriched KEGG pathways (C) and GO annotations(D) of METTL3 co-expressing genes in LIHC. [file 12935_2023_3096_MOESM3_ESM.jpg]
